# Chitosan as a Functional Carrier for the Local Delivery Anti-Inflammatory Systems Containing *Scutellariae baicalensis radix* Extract

**DOI:** 10.3390/pharmaceutics14102148

**Published:** 2022-10-10

**Authors:** Magdalena Paczkowska-Walendowska, Judyta Cielecka-Piontek

**Affiliations:** Department of Pharmacognosy, Poznan University of Medical Sciences, Rokietnicka 3, 60-806 Poznan, Poland

**Keywords:** *Scutellariae baicalensis radix*, chitosan, antioxidant activity, anti-hyaluronidase activity

## Abstract

The aim of the study was to establish the influence of chitosan on the preparation of systems containing *Scutellariae baicalensis radix* extract and to demonstrate the potential of anti-inflammatory action for the treatment of periodontitis. In the first stage, the impact of the variables (extraction mixture composition, temperature, and the number of extraction cycles) on the extracted samples’ biological characteristics was analyzed using the Design of Experiments (DoE) approach. The best conditions for baicalin, baicalein, and wogonin extraction from *Scutellariae baicalensis radix* were 80% methanol in the extraction mixture, 70 °C, and 4 cycles per 60 min. The DoE approach can be used to choose the best chitosan system parameters with equal success. An increase in the deacetylation degree of chitosan used in the system improved the potential for reducing free radicals and inhibiting the hyaluronidase enzyme. Also, increasing the degree of chitosan deacetylation results in increased resistance of the carrier to biodegradation and an extended baicalin release profile, which is also associated with an increase in the viscosity of the chitosan-based system. In total, the system of a freeze-dried extract with chitosan 90/500 in the ratio of 2:1 (system S9) turns out to be the one with the best physicochemical (high percentage of baicalin release and the highest viscosity conditioning the prolonged stay at the site of administration) and biological properties (the highest antioxidant and anti-inflammatory activities), resulting in the highest potential for use in the treatment of oral inflammatory diseases.

## 1. Introduction

To increase the pro-health benefits of the plant material, its extracts can be combined with functional polymers with additional health-promoting properties. One such substance is chitosan [1]. Chitosan is a natural polycationic linear polysaccharide derived from the partial deacetylation of chitin and composed of β-(1-4)-linked D-glucosamine and N-acetyl-D-glucosamine randomly distributed within the polymer. Each chitosan is described by two parameters, the degree of deacetylation, and its viscosity. The degree of chitosan deacetylation greatly influences the polymer’s characteristics: charge density (the number of available primary amines for binding), solubility, crystallinity, and degradation rate [2]. Chitosan has specific, intriguing properties that make it suitable for usage in various biomedical applications, including biocompatibility, non-toxicity, minimal allergenicity, and biodegradability [3]. It can be used in pharmaceutical excipients or drug carriers [4], wound-healing materials [5], and as a scaffold for tissue engineering [6]. Chitosan is often used in pharmaceutical technology as an ingredient controlling and delaying the release of both synthetic [7,8,9] and natural ingredients [10,11].

Periodontal disease is a significant global oral health burden, and severe periodontitis accounts for multiple tooth losses in the adult population worldwide. Oral health and hygiene are impacted by conditions such as periodontitis, dental caries, illnesses of the mucosa and salivary glands, orofacial discomfort, and clefts [12]. According to the Global Burden of Disease 2015 report, the number of people with untreated oral diseases increased from 2.5 billion in 1990 to 3.5 billion in 2015, while the number of people with a disability-adjusted life year increased by 64% [13]. Periodontitis, a long-lasting inflammatory condition of the periodontal tissue brought on by a disruption in the control of the host’s inflammatory response to bacterial infection, was made worse by oxidative stress. In addition, it suggests that oral diseases are not caused by the overgrowth of a single pathogen, as previously thought, such as *Streptococcus mutans* in dental caries, but are due to a dysbiotic composition of the oral microbiome, which has been shown in comparison with healthy subjects [13]. When pathogens are engulfed by human leukocytes, reactive oxygen species (ROS) are also formed. Oxidative stress, brought on by proteins, lipids, and nucleic acids changing in structure and function, may be brought on by producing ROS associated with inflammation [14].

In recent years, much attention has been devoted to searching for novel herbal therapies with anti-inflammatory, antioxidant, and antibacterial properties for treating and preventing oral diseases [15,16]. One of the more exciting plant materials used in oral infections is *Scutellariae baicalensis radix* (Baikal Skullcap Root). Flavones such as baicalin, wogonoside, and their aglycones, baicalein and wogonin, are the major bioactive compounds extracted from the *S. baicalensis* root [17]. Due to its many favorable effects on inflammatory processes in the oral cavity, *S. baicalensis* radix has the potential to treat periodontal disorders. Inhibiting the expression of proinflammatory mediators such IL-1, IL-6, IL-8, and TNF in gingival tissues first lowers inflammation, slows the loss of alveolar bone, and encourages the regeneration of periodontal structures [18]. It is directly related to baicalin, baicalein, and wogonin in the plant material. *S. baicalensis* has an intense antibacterial activity against oral pathogens, including *Streptococcus mutans*, *S. Fusobacterium nucleatum*, *Aggregatibacter actinomycetemcomitans*, and *Porphyromonas gingivalis* [19]. In addition to anti-inflammatory and antibacterial properties, plant material also has an antioxidant effect, which is no less critical in the pathogenesis of oral diseases [20]. Combinations of *S. baicalensis* with chitosan have been described in the literature in the context of its use in the treatment of periodontal diseases [21] and vaginal infections [22]. Still, the topic has not been thoroughly understood, for example, from the influence of chitosan properties on the physicochemical parameters of the systems.

Considering the above, the conducted studies focused on the evaluation of the potential of chitosan in the development of a buccal anti-inflammatory delivery system containing *S. baicalensis radix* extract. For this purpose, for the first time, the influence of three types of chitosan differing in the degree of deacetylation was used to obtain binary mixtures. Additionally, the DoE approach was used for the first time to optimize extraction processes as well as for binary mixture preparation. The composition of the system and influence of the deacetylation degree of chitosan was assessed for biological activity (antioxidant and anti-hyaluronidase activities that may be involved in the treatment of periodontitis) as well as for the physicochemical properties of the systems (release of active substance and rheological properties related to the binding of the polymer to mucin).

## 2. Materials and Methods

### 2.1. Plant Material

Plant raw material, *Scutellariae baicalensis radix*, was purchased from NANGA (Zlotow, Poland), country of origin: China (Lot No. 243042021).

### 2.2. Chemicals and Reagents

Baicalin (≥95%, HPLC), baicalein (≥98%, HPLC), and wogonin (phyproof^®^ Reference Substance) were obtained from Sigma-Aldrich (Poznan, Poland). Excipients, such as chitosans with a degree of acetylation of 70%, 80%, and 90% with a viscosity range of 500 mPas (marked as 70/500, 80/500, and 90/500, respectively) were supplied from Heppe Medical Chitosan GmbH (Halle, Germany). Reagents for activity assays: 2,2-Diphenyl-1-picrylhydrazyl (DPPH), sodium chloride, bovine serum, hexadecyltrimethylammonium bromide (CTAB), hyaluronic acid (HA), for dissolution studies: potassium chloride, sodium chloride, di-potassium hydrogen orthophosphate, magnesium chloride, calcium chloride, and xylitol, and mucoadhesive tests: mucin from porcine stomach were obtained from Sigma-Aldrich (Poznan, Poland). HPLC grade acetonitrile and water were obtained from Merck. High-quality pure water and ultra-high-quality pure water were prepared using an Direct-Q 3 UV Merck Millipore purification system.

### 2.3. Obtaining and Characterizing Biological Activity of Scutellariae Baicalensis Radix Extract

#### 2.3.1. Plant Extraction Using Design of Experiment (DoE)

Using the Design of Experiments (DoE) approach, a factor experiment plan was developed for three independent variables, which were assigned three levels of values (3^2^ full factorial design). The content of the extraction mixture, its temperature, and the number of extraction cycles for 60 min were selected as independent factors (Table 1). The plant material was extracted using ultrasonic waves.

The parameters used to assess extraction efficiency were: the determination of baicalin content, total phenolic content, and antioxidant and anti-hyaluronidase activities.

#### 2.3.2. Determination of Selected Active Components Content and Total Phenolic Content (TPC)

The contents of the main active compounds (baicalin, baicalein, and wogonin) were determined by using the HPLC-Diode-Array Detection method. Separations were performed on a Kinetex^®^ C18 column, 5 μm particle size, 100 mm × 2.1 mm (Phenomenex, Poland). The detection was performed using a diode array detector at a wavelength maxima (λmax) of 280 nm. The mobile phase was composed of phosphoric acid 0.1% (A) and acetonitrile (B) with a gradient elution: 0–20 min, 10–40% B; 20–22 min, 10% B. The flow rate of the mobile phase was 1.0 mL/min, and the column temperature was maintained at 30 °C.

The total content of phenolic components was determined by using the method described previously [23]. Briefly, 25 µL of the extracts or gallic acid solution (in concentration range 6.25–100 μg/mL), 200 µL of distilled water, 15 µL of Folin-Ciocalteu reagent, and 60 µL of 20% sodium carbonate solution were added. The plate was shaken for 5 min at 600 rpm, and incubated for a further 25 min at room temperature in the dark. Absorbance was measured at 760 nm (Multiskan GO 1510, Thermo Fisher Scientific, Vantaa, Finland). The test was repeated six times. Using the calibration curve for gallic acid, the total gallic acid content in the prepared extracts was calculated and expressed as milligrams of gallic acid equivalents (GAE) per 1 g of plant material.

#### 2.3.3. Determination of Biological Activity

##### Antioxidant Activity

Antioxidant activity was determined using an assay with 2,2-Diphenyl-1-picrylhydrazyl (DPPH), according to the previously described procedure [23]. Briefly, 25 μL of extracts were mixed with a 175 μL of 0.2 mmol/L DPPH solution. The reaction mixture was shaken and incubated in the dark at room temperature for 30 min. Compared to the blank (25 μL of the extraction mixture and 175 μL of methanol), absorbance was measured at 517 nm (Multiskan GO 1510, Thermo Fisher Scientific, Vantaa, and Finland). 25 μL of the extraction mixture and 175 μL of the DPPH solution made up the control sample. Each assay was carried out six times. The formula used to determine the % of DPPH scavenging activity is as follows:

$${\mathrm{DPPH}}_{\mathrm{scavenging}\;\mathrm{activity}\;}\left(\%\right)=\frac{A_{0}-A_{1}}{A_{0}}\times 100\%$$

where A_0_ is the absorbance of the control, and A_1_ is the absorbance of the sample.

##### Anti-Hyaluronidase Activity

The procedure of hyaluronidase inhibition was determined by the previous turbidimetric method [23]. Briefly, reagent mixtures were created by mixing 25 µL of hyaluronidase enzyme (30 U/mL of acetate buffer pH 7.0), 25 µL of acetate buffer (50 mM, pH 7.0, with 77 mM NaCl, and 1 mg/mL of bovine albumin), 15 µL of acetate buffer (pH 4.5), and 10 µL of extracts. For 10 min, all of the reaction mixtures were incubated at 37 °C. Hyaluronic acid solution (HA; 0.3 mg/mL of acetate buffer pH 4.5) was then added, and the mixture was incubated at 37 °C for 45 min. To precipitate the undigested HA, 200 µL of 2.5% CTAB in 2% NaOH was added. The mixture was left at room temperature for 10 min. The turbinance of the reaction mixture was measured as the absorbance at 600 nm (Multiskan GO 1510, Thermo Fisher Scientific, Vantaa, Finland).

Also, 5 blank samples have been prepared:

- blank 1 (B1): enzyme and hyaluronic acid solution were replaced with acetate buffer (25.0 µL), and the extract was replaced with the extraction mixture (10.0 µL),
- blank 2 (B2): the enzyme solution was replaced with acetate buffer (25.0 µL), and the extract was replaced with the extraction mixture (10.0 µL),
- blank 3 (B3): the extract was replaced with the extraction mixture (10.0 µL),
- blank 4 (B4): hyaluronic acid solution replaced with acetate buffer (25.0 µL),
- blank 5 (B5): the enzyme solution was replaced with acetate buffer (25.0 µL).

The following equation was used to calculate the percentage of hyaluronidase inhibition:

$$I\%=\frac{\left(A_{S}-A_{B4}\right)-\left(A_{B3}-A_{B1}\right)}{\left(A_{B5}-A_{B4}\right)-\left(A_{B3}-A_{B1}\right)}\times 100\%$$

where: I%—% inhibition of hyaluronidase, A_S_—absorbance of the sample, A_B1_—absorbance of blank 1, A_B3_—absorbance of blank 3, A_B4_—absorbance of blank 4, A_B5_—absorbance of blank 5.

### 2.4. Preparation and Characterization of Systems with Chitosan

#### 2.4.1. Preparation of a Freeze-Dried Extract

The optimized E10 extract was prepared in accordance with the following conditions: 80% methanol in the extraction mixture, temperature 70 °C, and 4 cycles for 60 min. Then the extract was frozen and lyophilized (CHRIST 1–4 LSC, Osterode am Harz, Germany). The temperature on the freeze dryer shelf was heated and ranged from +15 °C to +20 °C, the temperature inside the product estimated −4 °C and the condensation temperature was set to −48 °C. The freeze-drying was conducted at reduced pressure (1.030 mbar) for 48 h.

The content of active compounds (baicalin, baicalein, and wogonin) in the lyophilized extract was determined using the HPLC method described above.

#### 2.4.2. Optimization of Chitosan Systems

To optimize the preparation of the buccal delivery system, the Design of Experiment (DoE) approach was used. The factor experiment design was developed for two independent variables that were assigned three levels of values (3^2^ full factor design). The degree of chitosan deacetylation and the ratio of chitosan and lyophilized extract were selected as independent factors (Table 2). For example, the S1 system was prepared by weighing chitosan 70/500 into an agate mortar and then adding the freeze-dried extract at a weight ratio of 2:1. The system was kneaded to obtain a homogeneous mixture. The system was stored at room temperature at ambient conditions.

#### 2.4.3. The Identification of Optimized Systems

Fourier Transform Infrared Spectroscopy with Attenuated Total Reflectance (FTIR-ATR)

The FTIR-ATR spectra were measured between 400 cm^−1^ and 4000 cm^−1^, with a res-olution set to 1 cm^−1^, with a Shimadzu IRTracer-100 spectrometer equipped with a QATR-10 single bounce-diamond extended range and LabSolution IR software (Warsaw, Poland).

#### 2.4.4. Determination of Biological Activity

Antioxidant and anti-hyaluronidase activities were performed according to the procedures described above.

#### 2.4.5. Dissolution Studies of Active Compound

Dissolution studies of active compound (baicalin) from lyophilized extract as well as chitosan systems were performed using an Agilent 708-DS dissolution apparatus. A standard basket method was used at 37 ± 0.5 °C with a stirring speed of 50 rpm. Samples were weight into gelatine capsules and then placed in 300 mL of artificial saliva solution at pH 6.8 (potassium chloride (1.20 g), sodium chloride (0.85 g), di-potassium hydrogen orthophosphate (0.35 g), magnesium chloride (0.05 g), calcium chloride (0.20 g), xylitol (20.0 g) and water up to 1 L; pH was adjusted to 6.8 by 1 M HCl). The liquid samples were collected at specified time intervals, and an equal volume of temperature-equilibrated media was replaced. The samples were filtered through a 0.45 μm nylon membrane filter. The concentrations of baicalin, baicalein, and wogonin in the filtered acceptor solutions were determined by the HPLC method described above. Sink conditions were preserved in the studies.

#### 2.4.6. In Vitro Assessment of Mucin-Biopolymer Bioadhesive Bond Strength

A viscometric method was used to quantify mucin-polymers’ bioadhesive bond strength. The evaluation was performed according to the method described previously [24]. Briefly, the viscosity coefficient of a hydrophilic dispersion including mucin and chitosan systems was calculated using the equation below:

η_t_ = η_m_ + η_p_ + η_b_

where η_t_ is the viscosity coefficient of the system, and η_m_ and η_p_ are the individual viscosity coefficients of mucin and bioadhesive polymer, respectively, and η_b_ is the viscosity of component due to bioadhesion and can be obtained by rear-ranging above equation:

η_b_ = η_t_ − η_m_ − η_p_

The force of bioadhesion F, represents the additional intermolecular frictional force per unit area and was determined by:

F = η_b_σ

where σ is the rate of shear per second.

### 2.5. Statistical Analysis

Statistical analysis was carried out with Statistica 13.3 software (TIBCO Software Inc., Palo Alto, CA, USA). The normality of the results was checked using the Shapiro–Wilk test. The differences among the mean values were tested using the ANOVA test with post hoc Tukey’s range test for multiple comparisons. Differences between groups were considered to be significant at *p* < 0.05.

## 3. Results

The use of complementary and herbal medicines for treating and preventing many disorders has recently gained popularity. A critical element in preparing the product is an appropriate extraction process. Only effective extraction and standardization of the obtained product can ensure biological effectiveness and safety. Therefore, more and more attention is paid to conducting the extraction process following the Design of Experiments (DoE) approach, thanks to which complete information about the process is obtained with a minimized number of experiments. Therefore, the first stage of the research was to plan the investigation by the DoE with a full factorial design model, where the input factors were the percentage of methanol in the extraction mixture, its temperature, and the number of process cycles (Table 1).

**Figure 1:**
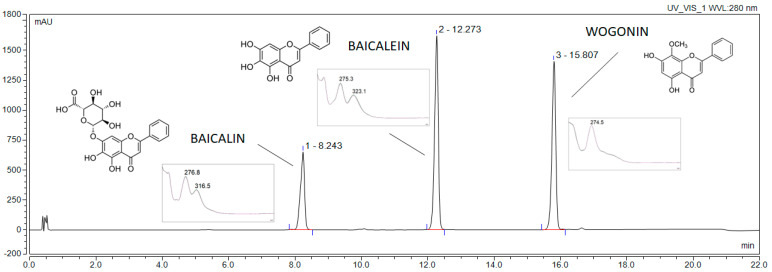
Chromatogram of standards.

Firstly, all extracts were tested for total polyphenol content (Table 3). The HPLC-DAD method was used to assess the qualitative and quantitative composition in regards to baicalin, baicalein, and wogonin (Figure 1).

The content of baicalin, baicalein, and wogonin in the produced E1–9 extracts may be qualitatively measured using the above-mentioned HPLC-DAD method. Table 3 compiles the active compound content.

**Figure 2:**
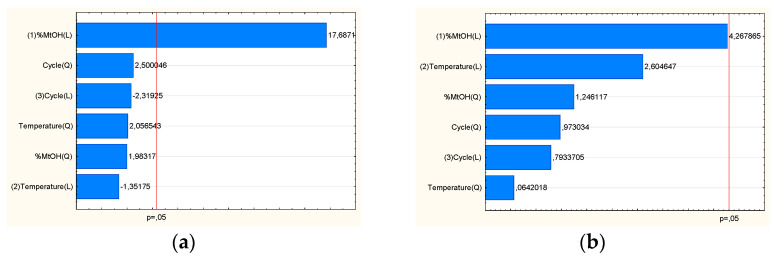
Pareto plot of standardized effects for the baicalin content (**a**) and TPC (**b**).

The baicalin content in the extracts was the first output parameter of the DoE model. Based on the Pareto diagram (Figure 2a), it can be indicated that the percentage of methanol in the extraction mixture is a statistically significant factor affecting the content of baicalin in the extract. This effect has a positive sign i.e., the baicalin content rises as the proportion of methanol in the extraction mixture increases. Then, the TPC value was considered. The amount of methanol in the extraction mixture is a statistically significant factor influencing the content of the TPC, according to the Pareto diagram (Figure 2b).

It is crucial to ascertain the biological activity of the produced extracts to evaluate the impact of extraction parameters on their effectiveness. Due to their antioxidant capabilities, several herbal extracts or organic substances are crucial in treating different oral disease symptoms. Free radicals and reactive oxygen species are critical in the pathophysiology of oral diseases because they boost the inflammatory response. Although the antioxidant activity of *S. baicalensis radix* extracts is well known, it was necessary to check the input parameters’ influence by using the DPPH free radical scavenging method. Moreover, in oral diseases, hyaluronic acid is significantly depolymerized into fragments of lower molecular weight by the action of hyaluronidases, β-glucuronidases, hexosaminidases, and reactive oxygen species [25]. Patients with early periodontal disease have been shown to have gingival tissue that contains pro-inflammatory low molecular weight hyaluronate. Examining extracts’ ability to inhibit hyaluronidase was vital because natural substances and plant extracts are known to do so. The complete results for both antioxidant and anti-hyaluronidase activities are shown in Table 4.

**Figure 3:**
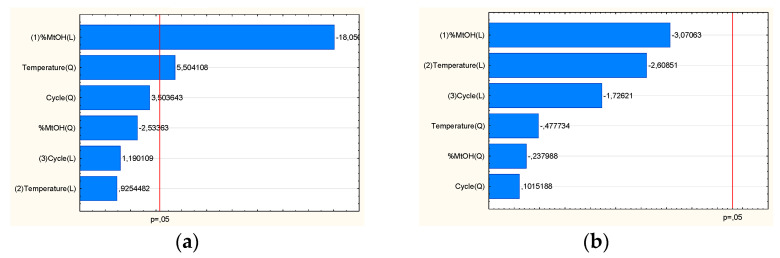
Pareto plot of standardized effects for the antioxidant activity using DPPH assay (**a**) and for anti-hyaluronidase activity (**b**).

Analyzing the data on antioxidant activity, it was shown that the E9 extract showed the highest activity. It was proven that the amount of methanol in the extraction mixture and temperature are statistically significant variables impacting the IC50 in the case of antioxidant activity (Figure 3a). The antioxidant activity of *S. baicalensis* radix has been widely known [26]. Flavones from *Scutellariae radix* have antioxidant action depending on structural characteristics, including the quantity and position of hydroxyl and methoxy groups and glycosylation. The presence of flavonoids with a significant number of phenolic hydroxyl groups plays a critical role in the scavenging activity of the *S. baicalensis radix* extract [27]. In an alkaline solution, baicalein, and baicalin can combine to create stable free radicals that may have an active location in the 6-OH group [28]. Direct antioxidant mechanisms, including radical scavenging, indicated in the literature, have also been proven in this work.

Hyaluronic acid is a naturally occurring glycosaminoglycan of the extra-cellular matrix of the oral cavity, which helps relieve pain and reduce gingival inflammation associated with oral lesions, dry mouth, ulcers, gingivitis, and other types of oral wounds. Hyaluronic acid plays a major role in periodontal tissue differentiation and proliferation and reduces local inflammatory processes. Moreover, hyaluronan can reduce the colonization and proliferation of pathogenic bacteria in the gingival crevice and adjacent periodontal tissues [29]. It was shown that the E9 extract showed the highest activity. Although none of the effects had a statistically significant impact on the inhibition of the hyaluronidase enzyme (Figure 3b), it can be seen that the increase in methanol content in the extraction mixture and temperature was influenced by the rise in anti-hyaluronidase activity expressed as a decrease in IC_50_ value. Literary data also indicate a strong influence of the plant material on inhibiting the hyaluronidase enzyme [30]. Using computational modeling approaches, it was possible to demonstrate that hydrophobic interaction, electrostatic force, and hydrogen bond are the main interaction forces between baicalin and hyaluronidase. The results show that the binding leads to changes in the secondary structure of the enzyme [31]. A significant increase in the anti-inflammatory effect, including the inhibition of the degradation of hyaluronic acid, indicates the combined effect of active compounds from *S. baicalensis* extract, particularly baicalin and baicalein [32].

**Figure 4:**
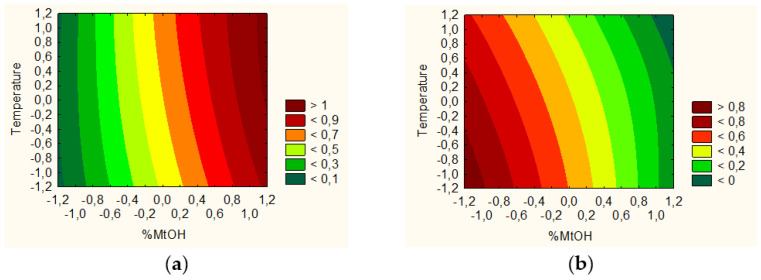
Model utility contour profiles for effect with a positive sign (**a**) baicalin content and TPC, and negative sign (**b**) antioxidant and anti-hyaluronidase activities.

It was feasible to identify technical aspects of the extraction process that would produce the extract with the best qualities and the maximum activity based on the findings of research and statistical analyses. TPC and the percentage of baicalin were shown to be significant input factors (Figure 4a), while antioxidant and anti-hyaluronidase activities were found to be considerable input factors (Figure 4b). The utility contour profiles allowed for the prediction of the model and the identification of the optimal extraction process parameters: 80% methanol in the extraction mixture, 70 °C, and 4 cycles (statistically insignificant parameter). Although there are reports of the use of other types of extractants and extraction methods (60% methanol [21] or 70% methanol using classical extraction [33]), it becomes necessary to use a statistical approach so that the processed extract is characterized by the highest biological activity for a given indication. The use of the DoE approach seems to meet these expectations.

The E10 extract was created using optimized process parameters, and its activity was assessed to validate the model. Then, the E10 extract was lyophilized to obtain a solid extract which was used to prepare systems with chitosan. Chitosan has been selected as one of the excipients with potent anti-inflammatory properties [12]. While the effect of chitosan molecular weight on anti-inflammatory activity has been investigated [34], no data are available on the impact of chitosan deacetylation. Therefore, according to the DoE approach, the effect of the degree of deacetylation of chitosan and its percentage in the system was investigated in this study (Table 2).

**Figure 5:**
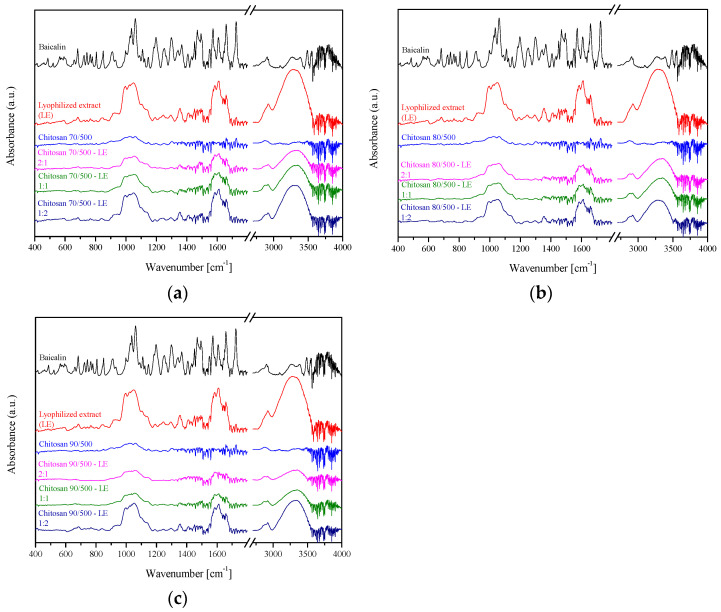
FTIR-ATR spectra of baicalin, *S. baicalensis radix* extract and its systems with chitosan 70/500 (**a**), chitosan 80/500 (**b**) and chitosan 90/500 (**c**). Characteristic bands of *S. baicalensis* lyophilized extract at 3330 cm^−1^, 1720 cm^−1^, and 1660 cm^−1^ were connected with the stretching vibration of the O–H, –COOH, and C=O groups, where signals at 1600 cm^−1^ and 1580 cm^−1^ with the C=C vibration stretching of the aromatic rings in the structure of flavones. The broad bands in the range 1200–900 cm^−1^ were connected to the various stretching vibrations of C–O bonds of saccharides [21]. Described wavelengths were also those characteristics of baicalin, the main active compound in the extract [35]. When analyzing spectra of chitosan, one can notice characteristic bands at 3360 cm^−1^ and 3300 cm^−1^ bands coming from stretching vibrations of O-H and N-H groups, at 2900 cm^−1^ from C-H vibrations, and at 1650 cm^−1^ from N-H vibrations. Spectra of chitosans with different degrees of deacetylation do not differ in terms of the position of individual bands. However, quantitative IR analysis may be used to characterize the exact degree of deacetylation [36]. Finally, when analyzing spectra for the systems, it can be observed that the spectra are the sum of the spectra for individual components such as lyophilized extract and chitosan. Thus, it can be concluded that the prepared systems are physical mixtures, and no changes in the chemical structure of the *S. baicalensis* extract occurred after the system preparation process. Therefore, it is the method of preparing the system that has a decisive impact on the formation of connections between the components, and grinding leads to the formation of only a physical mixture [21], while in the case of other preparation methods, ATR spectra may indicate interactions between the extract and chitosan [22].

The obtained chitosan systems were characterized in terms of their possible intermolecular chemical bond formation (FTIR-ATR) and presented in Figure 5.

The scavenging rate of systems S1–S9 samples increaseda with increasing concentration of extract (Table 5), whose antioxidant activity is much greater than that of chitosan. Chitosan has negligible radical scavenging activity due to insufficient H-atoms donors [37], but its activity, although slight, is due to the action of nitrogen on the C–2 position of chitosan. Xia et al. suggest the scavenging mechanism of chitosan, where the nitrogen of amino groups has a lone pair of electrons, which can attach to a proton released from an acidic solution to form ammonium (NH_3_^+^) groups. The hydrogen ion from the NH_3_^+^ may react with the free radicals to create a stable molecule [38]. So, analyzing the results for chitosan alone, also based on the Pareto plot (Figure 5a), the number of free amino groups is essential to excellent antioxidant performance since a high degree of deacetylation resulted in chitosan with better antioxidant properties.

**Figure 6:**
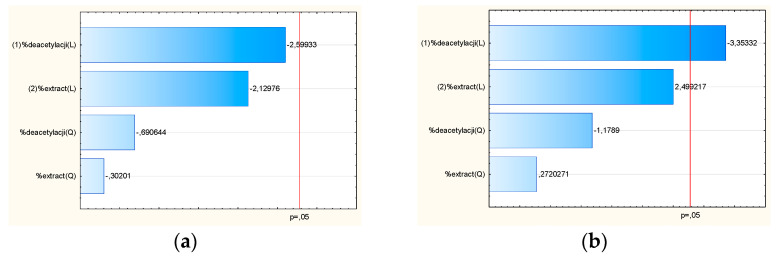
Pareto plot of standardized effects for the antioxidant activity using DPPH assay (**a**) and for anti-hyaluronidase activity (**b**) for chitosan systems.

Analyzing data on anti-hyaluronidase activity (Table 5) shows that chitosan activity is much higher than extract. The inhibition of hyaluronidase was shown to be dependent on the concentration of chitosan in the system, so systems in a 2:1 ratio (chitosan-lyophilized extract) showed more activity (Figure 6b). It was also proven that the degree of deacetylation is a statistically significant variable impacting the IC_50_ in the case of anti-hyaluronidase activity (Figure 6b). The mechanism of inhibiting the activity of hyaluronidase may be related to the interaction of an increasing number of −NH_2_ groups with an increase in the deacetylation degree, which may lead to changes in the secondary structure of the enzyme [31]. Thus, a considerable increase in the inhibition of hyaluronic acid degradation may point toward a combined effect of active compounds from *S. baicalensis* extract, particularly baicalein and chitosan molecules [39]. A similar result was obtained by Mao et al. for chitosan oligosaccharide modified by grafting linalool [40].

**Figure 7:**
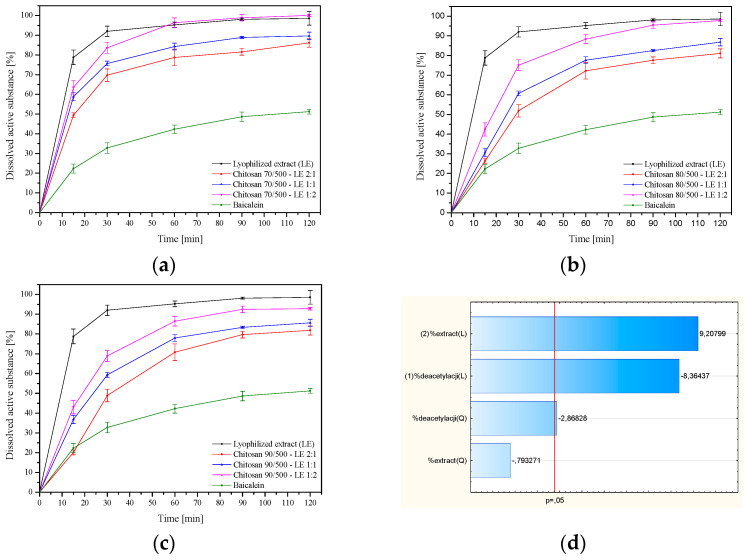
Dissolution profiles of baicalin from systems with chitosan 70/500 (**a**), chitosan 80/500 (**b**), and chitosan 90/500 (**c**), and Pareto plot of standardized effects for percentage of dissolved baicalin at 30 min (**d**).

To obtain a prolonged effect of a product in the oral cavity, a prolonged release of active compounds over time becomes essential. The compound with the highest content is baicalin (baicalin content: 2610.24 ± 0.68, baicalein: 323.40 ± 0.14, and wogonin: 43.10 ± 0.01 µg per 100 mg of lyophilized extract) and it was chosen as the marker for the release studies. Dissolution profiles are shown in Figure 7a–c.

The initial burst release, although small due to the low solubility of chitosan in alkaline pH, was attributed to the diffusion of the drug due to rapid swelling and was also partially related to the active substance adsorbed on the surface. It was found that as the percentage of chitosan in the system increases, the dissolution rate of the reference substances decreases (Figure 7d). Baicalin may dissolve to a lesser extent in dissolution media due to interactions between its hydroxyl group and the amino groups of chitosan, as well as van der Waals interactions. The cause could be the hydration of the system’s outer layer, which causes a gel layer to form on its surface [41]. This reduces the amount of water that enters the system’s core, which may obstruct the movement of reference compounds and cause their slow dissolution [42]. Therefore, less water may have permeated into the system’s core, leading to the active components’ progressive disintegration in this study. It has also been proven that an increase in the degree of chitosan deacetylation reduces the release of active compounds (Figure 7d). Luo et al. showed that increasing the degree of deacetylation of chitosan from 60.7% to 98.5% leads to enhanced biodegradation resistance of the carrier and prolonged release profile of the drug [43]. It is worth noting that the method of preparation of the system may significantly affect the release of baicalin. Chanaj-Kaczmarek et al. added solutions of lyophilized extract to 1% acetic acid solutions of chitosan (80% deacetylation degree and viscosity of 500 mPas and 1000 mPas) in a weight ratio 2:1, 1:1, and 1:2, obtained a different and extended-release profile of baicalin from the system. The increase in viscosity (500 vs. 1000 mPas) additionally slowed down the release of the active substance [22].

The dissolving data obtained for all chitosan systems were fitted to the following release models: zero-order, first-order, the Korsmeyer-Peppas model, the Higuchi model (used for the matrix systems), and (employed for the swellable matrices) (Table S1, Supplementary Materials). The greatest fit was obtained with the Higuchi model, which suggests that the baicalin was primarily released by diffusion and that its release was from a homogeneous flat matrix that did not degrade. To better comprehend the mechanism of baicalin release, it was important to look at the diffusional exponent *n* for the Korsmeyer-Peppas kinetic (*n* in the range 0.80–0.84), which characterizes the release as anomalous (for values 0.43 < *n* < 0.89) and suggests that the interaction of diffusion and erosion contributes to the control of active substance release. Chitosan can undoubtedly be considered a substance for the controlled release of substances with biological activity [44].

An important factor determining the application properties of a buccal product is the ability to bind the polymer to the mucin located in the mucosa. For this purpose, rheological studies of chitosan systems were used, and the results are presented in Figure 7.

**Figure 8:**
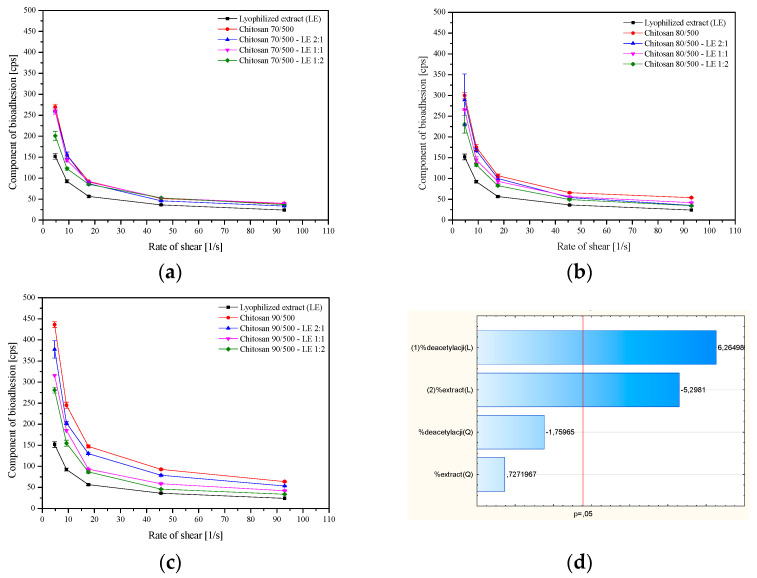
Component of bioadhesion of systems with chitosan 70/500 (**a**), chitosan 80/500 (**b**), and chitosan 90/500 (**c**), and Pareto plot of standardized effects for the component of bioadhesion (**d**).

According to the results presented in Figure 8a–c, it can be seen that the component of bioadhesion has a relationship: chitosan 90/500 > chitosan 80/500 > chitosan 70/500 and their respective systems. It can also be seen that the mucoadhesive properties of the entire system decrease with the increase of the extract content in the system. This is in line with the Pareto diagram analysis that the degree of deacetylation and content of extract are statistically significant factors affecting components of bioadhesion (Figure 8d). Previous studies also demonstrated that the interactions of chitosan–mucin in solution are significantly influenced by the degree of acetylation [45]. It is known that chitosan–mucin interacts mainly electrostatically, supported by other types of interactions (e.g., hydrogen bonds and hydrophobic association). High-Mw and high-degree deacetylation chitosan can interact with the same magnitude in a larger composition range compared to low-degree deacetylation chitosan, whose maximum interaction occurs only at a narrow range of composition [45]. Huang et al. discovered that decreasing polymer Mw and degree of deacetylation lowered the binding affinity and absorption capacity of chitosan molecules in solution and nanoparticles. Due to the electrostatic interactions between the positively charged chitosan and negatively charged mucus glycoproteins, it was also shown that the degree of deacetylation on mucoadhesion was more prevalent than the influence of Mw [46]. Additionally, Collado-González et al. indicated that the work on chitosan mucoadhesion to date showed that the degree of deacetylation is inversely proportional to the bioavailability of the substances contained in the complexes. The higher the deacetylation degree, the less homogeneously distributed the complexes in the mucosa due to the higher viscosity and, consequently, the lower the bioavailability of the drugs. The research results presented in this paper are consistent with these indications [47].

**Figure 9:**
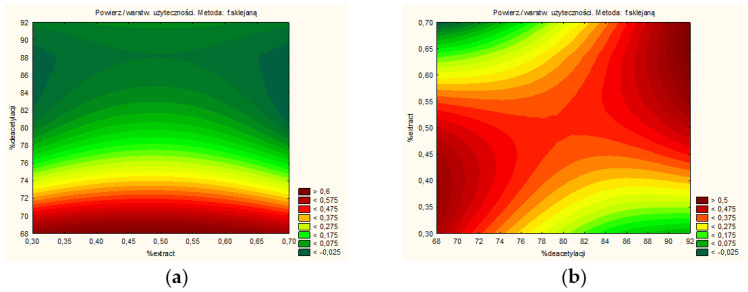
Model utility contour profiles for chitosan systems for effect with a negative sign (**a**) antioxidant and anti-hyaluronidase activities, and positive sign (**b**) release of baicalin and component of bioadhesion.

Finally, it was possible to predict the DoE model and select the parameters of the best system (Figure 9). Based on the utility contour profiles model, a system of the freeze-dried extract with chitosan 90/500 in the ratio of 2:1 was selected (system S9).

## 4. Conclusions

As a result of the application of the design of experiment (DoE) approach, it was possible to optimize the obtaining of an extract from *S. baicalensis radix* and prepare its systems with chitosan. It was shown that the percentage of methanol in the extraction mixture and the temperature were significantly important parameters that influenced the extraction efficiency of active compounds, such as baicalin from the *S. baicalensis radix.* The binary system containing lyophilized extract with chitosan 90:500 in weight ratio 2:1 was found to be most valuable with the appropriately controlled baicalin release, significant biological activity to inhibit the activity of the hyaluronidase enzyme, and appropriate mucoadhesive properties enabling prolonged residence time of the product at the application site. All the above properties of the chitosan system confirm the appropriate approach to the search for therapeutic solutions in the treatment of periodontal diseases based on the use of the anti-inflammatory potential of plant materials.

## Figures and Tables

**Table 1:** Factorial Extraction Process Experiment Plan.

	% of Methanol in The Extraction Mixture	Temperature [°C]	Number of Extraction Cycles
E1	40	30	2
E2	40	50	4
E3	40	70	3
E4	60	30	4
E5	60	50	3
E6	60	70	2
E7	80	30	3
E8	80	50	2
E9	80	70	4

**Table 2:** Factorial Experiment Plan for the optimization of chitosan systems.

	Degree of Deacetylation of Chitosan [%]	Ratio of Chitosan and Lyophilized Extract (*m/m*)
S1	70	2:1
S2	70	1:1
S3	70	1:2
S4	80	2:1
S5	80	1:1
S6	80	1:2
S7	90	2:1
S8	90	1:1
S9	90	1:2

**Table 3:** Factorial Extraction Process Experiment Plan.

	Baicalin [mg/20 mL Extract]	Baicalein [µg/20 mL Extract]	Wogonin [µg/20 mL Extract]	TPC [mg GAE/1 g Plant Material]
E1	139.00 ± 7.80	507.16 ± 65.53	45.28 ± 5.70	108.90 ± 4.69
E2	103.93 ± 4.00	193.90 ± 0.83	17.69 ± 7.63	127.18 ± 2.62
E3	239.50 ± 14.73	161.50 ± 10.41	24.70 ± 1.50	145.10 ± 4.76
E4	237.58 ± 20.78	323.84 ± 34.93	46.02 ± 4.90	146.22 ± 5.74
E5	312.89 ± 80.82	455.71 ± 22.14	53.75 ± 2.44	149.05 ± 1.79
E6	246.97 ± 19.74	370.00 ± 31.01	44.64 ± 3.72	152.52 ± 2.22
E7	377.11 ± 18.17	597.73 ± 68.36	72.87 ± 8.73	150.37 ± 7.24
E8	382.36 ± 10.14	575.03 ± 37.99	73.14 ± 3.60	157.41 ± 5.43
E9	331.34 ± 10.21	469.01 ± 23.73	56.61 ± 2.88	161.87 ± 5.67

**Table 4:** Antioxidant and anti-hyaluronidase activities of *S. baicalensis radix* extracts.

	DPPH IC_50_ [µg/mL]	Ability to Inhibit the Enzyme Hyaluronidase IC_50_ [mg/mL]
E1	100.73 ± 2.98	44.43 ± 1.00
E2	121.63 ± 3.48	24.97 ± 0.62
E3	119.69 ± 0.83	24.12 ± 0.45
E4	71.38 ± 0.14	23.76 ± 0.61
E5	92.92 ± 3.02	26.43 ± 1.35
E6	66.28 ± 3.23	22.12 ± 0.45
E7	52.61 ± 1.89	24.26 ± 0.70
E8	62.14 ± 2.66	18.15 ± 0.77
E9	47.96 ± 2.02	12.96 ± 0.56
Baicalin	19.04 ± 0.93	1.21 ± 0.06

**Table 5:** Antioxidant and anti-hyaluronidase activities of chitosan systems.

	DPPH IC_50_ [µg/mL]	Ability to Inhibit the Enzyme Hyaluronidase IC_50_ [mg/mL]
Lyophilized extract	15.22 ± 0.61	14.81 ± 0.67
S1	784.80 ± 20.60	3.41 ± 0.14
S2	255.28 ± 15.36	17.49 ± 0.43
S3	78.36 ± 3.24	24.63 ± 1.14
S4	253.76 ± 5.75	0.31 ± 0.02
S5	93.97 ± 3.67	4.69 ± 0.22
S6	36.01 ± 1.34	7.28 ± 0.27
S7	32.01 ± 1.56	0.41 ± 0.02
S8	23.70 ± 0.85	0.74 ± 0.04
S9	19.46 ± 0.98	3.04 ± 0.16
Baicalin	19.04 ± 0.93	1.21 ± 0.06
Chitosan 70/500	1542.95 ± 12.57	0.72 ± 0.03
Chitosan 80/500	1354.89 ± 13.75	0.34 ± 0.02
Chitosan 90/500	1014.05 ± 9.87	0.19 ± 0.01

## Data Availability

Not applicable.

## Supplementary Materials

The following supporting information can be downloaded at: https://www.mdpi.com/article/10.3390/pharmaceutics14102148/s1, Table S1: Mathematical characteristics of the baicalin release kinetics from chitosan systems.

## References

[1] PMC Chitosan: An Update on Potential Biomedical and Pharmaceutical Applications. https://www.ncbi.nlm.nih.gov/pmc/articles/PMC4557018/.

[2] Kiang T., Wen J., Lim H.W., Leong K.W. (2004). The effect of the degree of chitosan deacetylation on the efficiency of gene transfection. Biomaterials.

[3] PMC Biomedical Applications of Chitosan and Its Derivative Nanoparticles. https://www.ncbi.nlm.nih.gov/pmc/articles/PMC6415442/.

[4] PMC Chitosan-Based Nanomaterials for Drug Delivery. https://www.ncbi.nlm.nih.gov/pmc/articles/PMC6222903/.

[5] Frontiers Chitosan-Based Functional Materials for Skin Wound Repair: Mechanisms and Applications. https://www.frontiersin.org/articles/10.3389/fbioe.2021.650598/full.

[6] Sultankulov B., Berillo D., Sultankulova K., Tokay T., Saparov A. (2019). Progress in the Development of Chitosan-Based Biomaterials for Tissue Engineering and Regenerative Medicine. Biomolecules.

[7] Szymańska E., Woś-Latosi K., Jacyna J., Dąbrowska M., Potaś J., Markuszewski M.J., Winnicka K. (2020). The Correlation between Physical Crosslinking and Water-Soluble Drug Release from Chitosan-Based Microparticles. Pharmaceutics.

[8] Shahid N., Erum A., Zaman M., Tulain U.R., Shoaib Q., Malik N.S., Kausar R., Rashid A., Rehman U. (2022). Synthesis and Evaluation of Chitosan Based Controlled Release Nanoparticles for the Delivery of Ticagrelor. Des. Monomers Polym..

[9] Sip S., Paczkowska-Walendowska M., Rosiak N., Miklaszewski A., Grabańska-Martyńska K., Samarzewska K., Cielecka-Piontek J. (2021). Chitosan as Valuable Excipient for Oral and Topical Carvedilol Delivery Systems. Pharmaceuticals.

[10] Mujtaba M., Khawar K.M., Camara M.C., Carvalho L.B., Fraceto L.F., Morsi R.E., Elsabee M.Z., Kaya M., Labidi J., Ullah H. (2020). Chitosan-Based Delivery Systems for Plants: A Brief Overview of Recent Advances and Future Directions. Int. J. Biol. Macromol..

[11] Paczkowska M., Chanaj-Kaczmarek J., Romaniuk-Drapała A., Rubiś B., Szymanowska D., Kobus-Cisowska J., Szymańska E., Winnicka K., Cielecka-Piontek J. (2020). Mucoadhesive Chitosan Delivery System with Chelidonii Herba Lyophilized Extract as a Promising Strategy for Vaginitis Treatment. J. Clin. Med..

[12] Bhattacharyya A., Chattopadhyay R., Mitra S., Crowe S.E. (2014). Oxidative Stress: An Essential Factor in the Pathogenesis of Gastrointestinal Mucosal Diseases. Physiol. Rev..

[13] PubMed Global, Regional, and National Prevalence, Incidence, and Disability-Adjusted Life Years for Oral Conditions for 195 Countries, 1990–2015: A Systematic Analysis for the Global Burden of Diseases, Injuries, and Risk Factors. https://pubmed.ncbi.nlm.nih.gov/28792274/.

[14] Džunková M., Martinez-Martinez D., Gardlík R., Behuliak M., Janšáková K., Jiménez N., Vázquez-Castellanos J.F., Martí J.M., D’Auria G., Bandara H.M.H.N. (2018). Oxidative Stress in the Oral Cavity Is Driven by Individual-Specific Bacterial Communities. NPJ Biofilms Microbiomes.

[15] Palombo E.A. (2011). Traditional Medicinal Plant Extracts and Natural Products with Activity against Oral Bacteria: Potential Application in the Prevention and Treatment of Oral Diseases. Evid.-Based Complement. Alternat. Med..

[16] Gościniak A., Paczkowska-Walendowska M., Skotnicka A., Ruchała M.A., Cielecka-Piontek J. (2021). Can Plant Materials Be Valuable in the Treatment of Periodontal Diseases? Practical Review. Pharmaceutics.

[17] Zhao Q., Chen X.-Y., Martin C. (2016). Scutellaria Baicalensis, the Golden Herb from the Garden of Chinese Medicinal Plants. Sci. Bull..

[18] Cui L., Feng L., Zhang Z.H., Jia X.B. (2014). The Anti-Inflammation Effect of Baicalin on Experimental Colitis through Inhibiting TLR4/NF-ΚB Pathway Activation. Int. Immunopharmacol..

[19] Leung K.C.-F., Seneviratne C.J., Li X., Leung P.C., Lau C.B.S., Wong C.-H., Pang K.Y., Wong C.W., Wat E., Jin L. (2016). Synergistic Antibacterial Effects of Nanoparticles Encapsulated with Scutellaria Baicalensis and Pure Chlorhexidine on Oral Bacterial Biofilms. Nanomaterials.

[20] PMC Purification and Antioxidant Activities of Baicalin Isolated from the Root of Huangqin (*Scutellaria baicalensis gcorsi*). https://www.ncbi.nlm.nih.gov/pmc/articles/PMC3602557/.

[21] Chanaj-Kaczmarek J., Osmałek T., Szymańska E., Winnicka K., Karpiński T.M., Dyba M., Bekalarska-Dębek M., Cielecka-Piontek J. (2021). Development and Evaluation of Thermosensitive Hydrogels with Binary Mixture of *Scutellariae baicalensis radix* Extract and Chitosan for Periodontal Diseases Treatment. Int. J. Mol. Sci..

[22] Chanaj-Kaczmarek J., Rosiak N., Szymanowska D., Rajewski M., Wender-Ozegowska E., Cielecka-Piontek J. (2022). The Chitosan-Based System with *Scutellariae baicalensis radix* Extract for the Local Treatment of Vaginal Infections. Pharmaceutics.

[23] Paczkowska-Walendowska M., Gościniak A., Szymanowska D., Szwajgier D., Baranowska-Wójcik E., Szulc P., Dreczka D., Simon M., Cielecka-Piontek J. (2021). Blackberry Leaves as New Functional Food? Screening Antioxidant, Anti-Inflammatory and Microbiological Activities in Correlation with Phytochemical Analysis. Antioxidants.

[24] Paczkowska-Walendowska M., Szymańska E., Winnicka K., Szwajgier D., Baranowska-Wójcik E., Ruchała M.A., Simon M., Cielecka-Piontek J. (2021). Cyclodextrin as Functional Carrier in Development of Mucoadhesive Tablets Containing Polygoni Cuspidati Extract with Potential for Dental Applications. Pharmaceutics.

[25] Moseley R., Waddington R.J., Embery G. (2002). Hyaluronan and Its Potential Role in Periodontal Healing. Dent. Update.

[26] Gao Z., Huang K., Yang X., Xu H. (1999). Free Radical Scavenging and Antioxidant Activities of Flavonoids Extracted from the Radix of *Scutellaria baicalensis* Georgi. Biochim. Biophys. Acta BBA—Gen. Subj..

[27] ScienceDirect Structure-Activity Relationship of Eight High Content Flavonoids Analyzed with a Preliminary Assign-Score Method and Their Contribution to Antioxidant Ability of Flavonoids-Rich Extract from *Scutellaria baicalensis* Shoots. https://www.sciencedirect.com/science/article/pii/S1878535217301491.

[28] Woźniak D., Dryś A., Matkowski A. (2015). Antiradical and Antioxidant Activity of Flavones from *Scutellariae baicalensis radix*. Nat. Prod. Res..

[29] Casale M., Moffa A., Vella P., Sabatino L., Capuano F., Salvinelli B., Lopez M.A., Carinci F., Salvinelli F. (2016). Hyaluronic Acid: Perspectives in Dentistry. A Systematic Review. Int. J. Immunopathol. Pharmacol..

[30] Bhatti M.Z., Karim A. (2021). Plant Natural Products: A Promising Source of Hyaluronidase Enzyme Inhibitors.

[31] Li X., Liu H., Yang Z., Duan H., Wang Z., Cheng Z., Song Z., Wu X. (2021). Study on the Interaction of Hyaluronidase with Certain Flavonoids. J. Mol. Struct..

[32] Li B.Q., Fu T., Gong W.H., Dunlop N., Kung H., Yan Y., Kang J., Wang J.M. (2000). The Flavonoid Baicalin Exhibits Anti-Inflammatory Activity by Binding to Chemokines. Immunopharmacology.

[33] Li H.-B., Jiang Y., Chen F. (2004). Separation Methods Used for *Scutellaria baicalensis* Active Components. J. Chromatogr. B Anal. Technol. Biomed. Life Sci..

[34] Chang S.-H., Lin Y.-Y., Wu G.-J., Huang C.-H., Tsai G.J. (2019). Effect of Chitosan Molecular Weight on Anti-Inflammatory Activity in the RAW 264.7 Macrophage Model. Int. J. Biol. Macromol..

[35] Haider M., Hassan M.A., Ahmed I.S., Shamma R. (2018). Thermogelling Platform for Baicalin Delivery for Versatile Biomedical Applications. Mol. Pharm..

[36] Kumirska J., Czerwicka M., Kaczyński Z., Bychowska A., Brzozowski K., Thöming J., Stepnowski P. (2010). Application of spectroscopic methods for structural analysis of chitin and chitosan. Mar. Drugs.

[37] Wu C., Wang L., Fang Z., Hu Y., Chen S., Sugawara T., Ye X. (2016). The Effect of the Molecular Architecture on the Antioxidant Properties of Chitosan Gallate. Mar. Drugs.

[38] Xia W., Liu P., Zhang J., Chen J. (2011). Biological Activities of Chitosan and Chitooligosaccharides. Food Hydrocoll..

[39] In-Vitro Hyaluronidase Inhibition Assay of Chitosan Extracted from Exoskeleton of Freshwater Edible Crab *Sartoriana spinigera*. https://www.cabdirect.org/globalhealth/abstract/20193431451.

[40] Mao S., Liu X., Xia W. (2021). Chitosan Oligosaccharide-g-Linalool Polymer as Inhibitor of Hyaluronidase and Collagenase Activity. Int. J. Biol. Macromol..

[41] Ko J.A., Park H.J., Hwang S.J., Park J.B., Lee J.S. (2002). Preparation and Characterization of Chitosan Microparticles Intended for Controlled Drug Delivery. Int. J. Pharm..

[42] ScienceDirect Controlled Release of Indomethacin by Chitosan-Polyelectrolyte Complex: Optimization and In Vivo/In Vitro Evaluation. https://www.sciencedirect.com/science/article/abs/pii/016836599390080O.

[43] Luo L.-J., Huang C.-C., Chen H.-C., Lai J.-Y., Matsusaki M. (2018). Effect of Deacetylation Degree on Controlled Pilocarpine Release from Injectable Chitosan-g-Poly(N-Isopropylacrylamide) Carriers. Carbohydr. Polym..

[44] Prabaharan M., Mano J.F. (2004). Chitosan-Based Particles as Controlled Drug Delivery Systems. Drug Deliv..

[45] Menchicchi B., Fuenzalida J.P., Bobbili K.B., Hensel A., Swamy M.J., Goycoolea F.M. (2014). Structure of Chitosan Determines Its Interactions with Mucin. Biomacromolecules.

[46] He P., Davis S.S., Illum L. (1998). In Vitro Evaluation of the Mucoadhesive Properties of Chitosan Microspheres. Int. J. Pharm..

[47] Collado-González M., González Espinosa Y., Goycoolea F.M. (2019). Interaction Between Chitosan and Mucin: Fundamentals and Applications. Biomimetics.

