# Supplementary material for: Chitosan as a Functional Carrier for the Local Delivery Anti-Inflammatory Systems Containing Scutellariae baicalensis radix Extract
Source: Pharmaceutics. 2022 Oct 10;14(10):2148. doi: 10.3390/pharmaceutics14102148 (PMC9611887; doi:10.3390/pharmaceutics14102148)
Supplement: Supplementary file 1 [file pharmaceutics-14-02148-s001.zip › pharmaceutics-1943002-supplementary.pdf]

Table S1. Mathematical characteristics of the baicalin release kinetics from chitosan systems

Formulation	Mathematical model							
	Zero-order kinetic		First-order kinetic		Higuchi kinetic		Korsmeyer-Peppas kinetic	
	K	R^2	K	R^2	K	R^2	R^2	n
Chitosan 70/500 – lyophilized extract (LE) 2:1	33.94	0.64	1.44	0.40	**16.11**	**0.86**	0.70	0.81
Chitosan 70/500 – LE 1:1	34.30	0.59	1.43	0.38	**16.87**	**0.82**	0.68	0.82
Chitosan 70/500 – LE 1:2	38.85	0.60	1.47	0.38	**18.96**	**0.83**	0.68	0.83
Chitosan 80/500 – LE 2:1	37.99	0.81	1.57	0.51	**15.87**	**0.93**	0.77	0.82
Chitosan 80/500 – LE 1:1	39.43	0.78	1.56	0.48	**16.88**	**0.92**	0.75	0.83
Chitosan 80/500 – LE 1:2	42.48	0.73	1.55	0.44	**18.94**	**0.90**	0.73	0.84
Chitosan 90/500 – LE 2:1	40.33	0.84	1.65	0.55	**16.27**	**0.94**	0.80	0.83
Chitosan 90/500 – LE 1:1	38.04	0.76	1.53	0.46	**16.60**	**0.92**	0.73	0.82
Chitosan 90/500 – LE 1:2	40.49	0.73	1.52	0.44	**18.10**	**0.90**	0.72	0.83
